# Recurrent evolution of extreme longevity in bats

**DOI:** 10.1098/rsbl.2018.0860

**Published:** 2019-04-10

**Authors:** Gerald S. Wilkinson, Danielle M. Adams

**Affiliations:** Department of Biology, University of Maryland, College Park 20742, MD, USA

**Keywords:** lifespan, hibernation duration, sexual dimorphism, phylogenetic generalized least squares

## Abstract

Bats live longer than similar-sized mammals, but the number of lineages that have independently evolved extreme longevity has not previously been determined. Here we reconstruct the evolution of size-corrected longevity on a recent molecular phylogeny and find that at least four lineages of bats have lifespans more than fourfold those of similar-sized placental mammals, with the ancestral bat projected to live 2.6 times as long. We then evaluate a series of phylogenetic generalized least-squares models containing up to nine variables hypothesized to influence extrinsic mortality. These analyses reveal that body mass and hibernation predict longevity. Among hibernators, longevity is predicted by the absolute value of the median latitude of the species range and cave use, while cave use and lack of sexual dimorphism predict longevity among non-hibernators. The importance of torpor in extending lifespan is further supported by the one lineage with extreme longevity that does not hibernate but exhibits flexible thermoregulation, the common vampire bat. We propose several potential mechanisms that may enable bats to live so long, and suggest that the ability to tolerate a wide range of body temperatures could be important for surviving viral or other pathogen infections.

## 1. Background

The oldest mammal yet reported is a 211 year-old bowhead whale [1]. While impressive, that lifespan is arguably not as extreme as a 31 year-old naked mole-rat [2] because, as with many life-history traits, lifespan is allometrically related to body mass among mammals [3]. Identifying species with extreme lifespan requires, therefore, size correction. One approach is to divide observed lifespan by predicted lifespan for a non-flying placental mammal of the same body mass to obtain a longevity quotient (LQ) [4,5]. By this approach, naked mole-rats and modern humans [6] have comparable lifespans (LQ = 4.5), which greatly exceed the whale (LQ = 1.8). However, some bats are much more extreme, such as *Myotis brandtii* (LQ = 8) [7]. Determining whether such extraordinary longevity has evolved more than once is important for determining if proposed adaptations for long lifespan derived from single species studies (e.g. [8,9]) generalize to more than one lineage.

Life-history theory [10,11] predicts that selection for long lifespan requires low extrinsic mortality [12]. Thus, species with extreme lifespan should have a lower risk of mortality from factors such as accidents, infectious disease or predation, than species with short lifespan. For example, flight has been suggested to reduce extrinsic mortality in bats [5,13], but flight has evolved so few times that phylogenetic analysis is not possible. Moreover, all bats fly, so flight cannot explain lifespan variation among bats.

Many factors potentially influence extrinsic mortality risk in bats. Choice of diurnal roost environment can affect several risk factors. For example, roosting in caves may afford better protection from inclement weather and predation than roosting in foliage. Similarly, roosting in large aggregations may reduce predation risk owing to dilution or increased vigilance. However, these potential benefits may be offset by increased risk of pathogen and parasite transmission. For example, larger colonies of several bat species have been impacted more by a recent deadly fungal disease than smaller colonies [14].

Diet could also influence mortality risk. Whether food is stationary and predictable, such as a fruit or floral resource, or mobile and must be hunted and captured, can influence predator exposure and accident risk. Intestinal microbiome composition can be influenced by diet and has been implicated in lifespan [15,16]. Short or prolonged bouts of torpor can reduce starvation risk during periods of food shortage, although individuals with reduced body temperatures may be less able to avoid predation. Initial studies failed to find evidence that hibernating bats live longer than non-hibernating bats [5,17], but recent studies have found that hibernation reduces mortality [18] and extends lifespan in bats and other mammals [18–20].

Bats have slow reproductive rates [21] with long prenatal development [22], small litters and large neonates [23]. The need to fly with offspring has been proposed to explain why females are larger than males in many species [24,25]. In some species where females are larger than males, males live longer than females [7]. Conversely, in some harem polygynous species [26], males are larger than females but females live longer [27], presumably because male mortality is elevated. These observations suggest that mortality risk may also be impacted by reproductive rate and sexual competition.

In this study, we address two questions. First, how many lineages of bats exhibit extreme longevity? Second, what extrinsic or life-history factors are associated with increased longevity in bats? This study differs from a previous study [19] in several ways. Rather than use a concatenated supertree for comparative analyses, here we use a recent phylogeny based on DNA sequence data from five nuclear and four mitochondrial genes [28]. In addition, 50% of the bat longevity records are new or updated. Finally, we consider sexual dimorphism in body size and data source in addition to body mass, reproductive rate, hibernation, latitude, cave use, aggregation size and diet as potential predictors of longevity using phylogenetic generalized least squares (PGLS) [29] and use an information theory approach to evaluate parameter importance.

## 2. Methods

To identify lineages where lifespan has increased, we reconstructed the longevity quotient (LQ), i.e. the ratio of observed to predicted longevity, with squared-change parsimony on a molecular phylogeny of 67 bats [28] using Mesquite v. 3.6 [30]. We obtained longevity and body mass records from AnAge, build 14 [31]. Given that maximum lifespan is an order statistic that is expected to increase by diminishing amounts as sample size increases [32] we used only acceptable quality records with medium or large sample sizes to minimize sample size bias [33]. We predicted bat longevity by least-squares regression, i.e. log_10_(longevity) = 0.5609 + log_10_(body mass) × 0.1868, from 804 non-flying placental mammals.

To identify variables that explain variation in bat longevity, we evaluated alternative multivariate models [34] that corrected for common ancestry using PGLS, as implemented in CAPER [29]. Because LQ correlates with both longevity and body mass, we included log_10_(body mass) as a variable for potentially predicting log_10_(longevity). We used the absolute value of the median latitude (hereafter ‘latitude') of the species' range as a proxy for annual temperature and hibernation duration because in rodents hibernation duration increases linearly with mean annual temperature [20]. We added an interaction between hibernation and latitude to allow for the possibility that latitude may not affect longevity in non-hibernators. Additional variables included cave use (yes/no), diet type (animal/plant material), number of offspring produced per year and log_10_(breeding aggregation size). We used sexual dimorphism in total body length (TL), as measured by log_2_ (male-TL/female-TL), to determine if sexual selection on body size contributes to variation in longevity. Trait values were obtained from the literature, museum collections (see the electronic supplementary material), personal observation or personal communication. Because bat longevity records come from either captive or wild animals, we included data source in the models to insure it would not bias results.

We measured the relative importance of the phylogeny in predicting each trait by calculating Pagel's *λ* for continuous variables and *D* for binary variables [35] and then fitted all possible models using PGLS [36]. We rank-ordered models by the corrected Akaike information criterion (AICc) and calculated Akaike weights to determine model strength. We used models within 4 AICc of the best-fitting model for model averaging and estimated weighted coefficients, confidence intervals and relative importance for each variable [34,37]. Because the interaction between hibernation and latitude had significant influence, to interpret effects of the remaining variables we split the data by hibernator/non-hibernator, and then repeated the analyses described above.

## 3. Results

**Figure 1.**
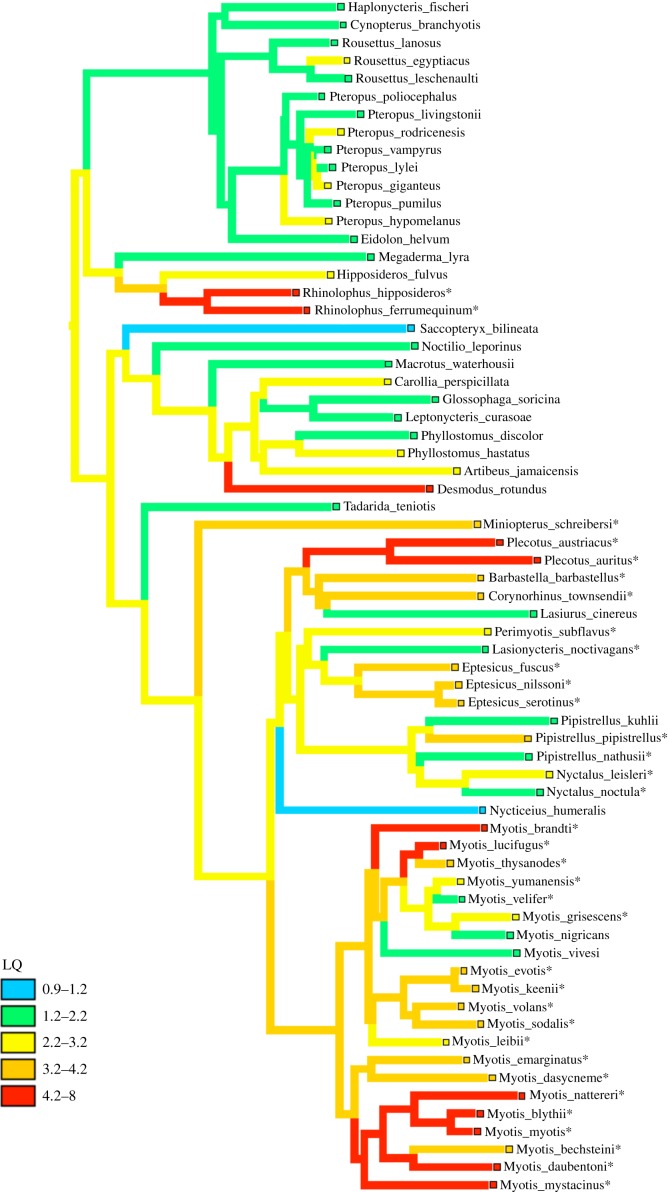
Ancestral state reconstruction by squared-change parsimony of longevity quotient (LQ) for bats, with * indicating hibernating species. (Online version in colour.)

Ancestral state reconstruction of longevity quotient (LQ) across the bat phylogeny reveals that extreme longevity, i.e. LQ > 4.2, has evolved at least four times (figure 1). The four lineages include horseshoe bats (genus *Rhinolophus*), a vampire bat (*Desmodus rotundus*), long-eared bats (genus *Plecotus*) and at least one *Myotis* lineage. Within *Myotis*, LQ has increased in several species, but decreased in others (figure 1). According to this reconstruction, the ancestral bat lived 2.64 times longer than a similar-sized placental mammal. Reconstruction of longevity expressed as a residual from the body size regression (not shown) identifies the same lineages.

Among the continuous variables hypothesized to influence longevity, all but aggregation size and progeny per year exhibit evidence of phylogenetic inertia, i.e. *λ* > 0 with *λ* ranging from 0.32 for sexual dimorphism to 0.88 for body mass (electronic supplementary material, table S1). Similarly, each of the three binary variables exhibits evidence of phylogenetic signal with *D* ranging from −0.16 for cave use to −1.0 for diet. Consequently, adjusting for phylogenetic covariance is necessary to evaluate variable importance in predicting maximum longevity.

**Figure 2.**
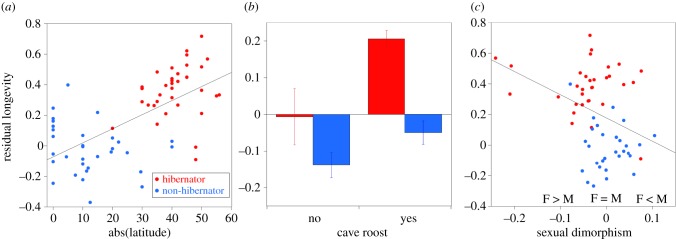
Relationships between residual longevity and predictive variables for hibernating (red) and nonhibernating (blue) species: (*a*) absolute value of median latitude, (*b*) cave use, and (*c*) sexual dimorphism in size (log_2_(male-TL/female-TL)). Residual longevities in (*a*) and (*c*) are from PGLS regressions of log_10_(longevity) on log_10_(body mass) + cave use, and in (*b*) from a PGLS regression of log_10_(longevity) on log_10_(body mass). Error bars indicate 1 s.e.m. (Online version in colour.)

Comparison of possible PGLS models revealed seven models within 4 AICc of the best-fitting model (electronic supplementary material, table S2). These models included four to six parameters and explained 63–69% of the variation in log longevity. Model averaging revealed that five variables had importance of 0.93 or greater, but only three variables had coefficients that differed from zero (electronic supplementary material, table S3). This apparent discrepancy was caused by a non-zero coefficient for the interaction between hibernation and latitude despite the coefficients on each of those two variables overlapping zero. Therefore, we split the data by hibernation and fitted separate sets of models to evaluate the relative importance of the remaining variables. These analyses revealed 10 models for hibernators and 14 models for non-hibernators within 4 AICc of the best-fitting model (table 1). The best-fitting hibernator model explained 46% of the variation in longevity while the best-fitting non-hibernator model explained 82%. Model averaging revealed that hibernator longevity is determined by body mass, latitude and cave use (table 2) with longevity increasing among species from more extreme latitudes (figure 2*a*). By contrast, model averaging revealed that non-hibernator longevity is determined by body mass, cave use and sexual dimorphism. Species that roost in caves have longer lifespans (figure 2*b*) while species in which males are larger than females have shorter lifespans (figure 2*c*). Hibernation is associated with greater longevity after controlling for each of the other explanatory variables (figure 2).

**Table 1.** Rank-ordered PGLS models within 4 AICc of the best model for predicting log_10_(longevity). Variables are *M* = log_10_(body mass), *L* = |median latitude|, *C* = cave use, *D* = sexual dimorphism, *A* = log_10_(aggregation size), *P* = progeny per year, *F* = diet, *S* = data source.

subset	model	AICc	ΔAICc	weight	*R*^2^
hibernator	*M* + *L* + *C*	−42.93	0	0.28	0.46
	*M* + *L* + *C* + *D*	−42.28	0.66	0.20	0.50
	*L* + *C* + *D*	−40.81	2.12	0.10	0.42
	*M* + *L* + *C* + *A*	−40.54	2.39	0.08	0.46
	*M* + *L* + *C* + *P*	−40.32	2.62	0.08	0.45
	*M* + *L* + *C* + *A* + *D*	−39.90	3.03	0.06	0.50
	*L* + *C* + *A* + *D*	−39.73	3.20	0.06	0.45
	*L* + *C* + *P* + *D*	−39.57	3.37	0.05	0.45
	*M* + *L* + *C* + *P* + *D*	−39.31	3.62	0.05	0.49
	*M* + *L*	−39.21	3.72	0.04	0.35
non-hibernator	*M* + *L* + *C* + *D*	−47.00	0	0.23	0.82
	*M* + *C* + *D*	−45.56	1.44	0.11	0.79
	*M* + *L* + *C* + *D* + *P*	−45.22	1.78	0.09	0.83
	*M* + *L* + *C* + *D* + *A*	−45.07	1.93	0.09	0.83
	*M* + *C* + *D* + *P*	−45.04	1.96	0.09	0.81
	*M* + *L* + *C* + *D* + *F*	−44.94	2.06	0.08	0.83
	*M* + *L* + *C* + *D* + *S*	−43.80	3.20	0.05	0.82
	*M* + *C*	−43.78	3.22	0.05	0.75
	*M* + *C* + *D* + *A*	−43.65	3.35	0.04	0.80
	*M* + *C* + *D* + *S*	−43.54	3.46	0.04	0.80
	*M* + *L* + *C*	−43.51	3.49	0.04	0.77
	*M* + *L* + *C* + *D* + *F* + *P*	−43.47	3.53	0.04	0.84
	*M* + *L* + *C* + *D* + *A* + *P*	−43.16	3.84	0.03	0.84
	*M* + *C* + *D* + *A* + *P*	−43.09	3.91	0.03	0.81

**Table 2.** Model-averaged conditional coefficients ± s.e. for models in table 1, with estimates ≠ 0 indicated in italics.

subset	variable	estimate (s.e.)	importance
hibernator	*intercept*	*0.739 ± 0.196*	
	*mass*	*0.223 ± 0.102*	*0*.*79*
	*latitude*	*0.012 ± 0.003*	*1*.*00*
	dimorphism	0.586 ± 0.368	0.52
	*cave use*	*−0.223 ± 0.092*	*0*.*96*
	progeny/yr	−0.059 ± 0.086	0.17
	aggregation size	0.030 ± 0.086	0.20
non-hibernator	*intercept*	*0.697 ± 0.093*	
	*mass*	*0.316 ± 0.040*	*1.00*
	latitude	−0.003 ± 0.002	0.64
	*dimorphism*	*−1.175 ± 0.504*	*0.92*
	*cave use*	*−0.136 ± 0.043*	*1.00*
	diet	−0.046 ± 0.045	0.12
	progeny/yr	0.052 ± 0.042	0.28
	aggregation size	−0.020 ± 0.020	0.19
	data source	−0.021 ± 0.050	0.09

## 4. Discussion

Several prior studies have reported that bats live longer than other mammals of similar body size [5,17–19]. However, the number of lineages in which longevity has increased could not be determined previously with confidence because of phylogenetic uncertainty. Our reconstruction of size-adjusted longevity (i.e. LQ) on a recent molecular phylogeny [28] reveals that extreme longevity has evolved at least four times in bats. This reconstruction also indicates that the ancestral bat could live 2.6 times as long as a placental mammal of similar body size—consistent with the expectation that the evolution of flight reduced the risk of extrinsic mortality for bats.

Species with extreme longevity also undergo hibernation in three out of four lineages of bats. The exception to this pattern is the common vampire bat, *Desmodus rotundus,* which can undergo torpor between feeding bouts [38]. In addition, female vampire bats live longer than males [39,40] and females that have been unsuccessful at obtaining a blood meal are more likely to receive food from a roostmate [41,42]. The number of potential food sharing partners likely reduces the risk of starvation [43,44], which almost certainly extends lifespan. Thus, like naked mole-rats [2,45], extreme longevity in vampire bats appears to co-occur with flexible thermoregulation and cooperative social behaviour.

PGLS analysis of longevity reveals similarities and differences with a previous study [19]. Both studies find that body mass, cave use and hibernation predict bat longevity but here latitude further predicts longevity among hibernators. From an evolutionary perspective, this is consistent with reduced extrinsic mortality in species that hibernate for longer durations. How lifespan is extended in hibernating species remains to be determined. One possibility is that reduced aerobic metabolism reduces oxidative damage. However, support for this once popular idea is equivocal or lacking [46,47], suggesting that other mechanisms may be involved.

Further evidence in support of mortality risk determining longevity was found in both studies by identifying cave use as an important predictor of longevity. In contrast to the earlier study [19], this study failed to find evidence that reproductive rate predicts longevity. We attribute this difference to more and better estimates of longevity along with little variation in reproductive rate among bats.

A final difference lies in the effect of sexual dimorphism, which was not considered before, but was an important predictor of longevity for non-hibernating species in this study. Interestingly, longevity is reduced for species in which males are larger than females (figure 2*c*). Most, if not all, of these species are polygynous and males fight for access to a group of females [26]. Among phyllostomid bats, males are larger than females in harem polygynous species [48]. Given that males fight, their reduced longevity is not unexpected. But, why females of those species also have reduced longevity is less obvious. Perhaps female survival is reduced by sexual conflict (e.g. [49]) due to aggression from dominant males or by birth of large infants.

Our phylogenetic comparative analyses identify five factors that explain variation in bat longevity and plausibly influence extrinsic mortality. However, the genetic and physiological mechanisms that enable some individuals to live longer than others remain to be elucidated. Records of neoplasms in bats are uncommon, but bats are not immune from cancer [50–55]. Nonetheless, no tumours have yet been reported for any species in the extreme lifespan lineages we identified. Thus, genetic adaptations for tumour suppression, which have been described for *Myotis brandtii* [8] and *Myotis myotis* [9], could contribute to extreme longevity. Moreover, recent studies have identified other possibilities, including improved DNA repair and immunocompetence [56], stabilization of microbiota [57], and reduced inflammation and viral tolerance or resistance enabled by flexible thermoregulation [58]. This latter idea is consistent with the important effect of hibernation found here and elsewhere [18,20]. Comparative genomic analyses using species that vary in size-adjusted longevity are needed to determine which of these potential mechanisms enable bats to live so long.

## Supplementary Material

Supplemental Methods

## Supplementary Material

Supplemental Table 1

## Supplementary Material

Supplemental Table 2

## Supplementary Material

Supplemental Table 3

## Data accessibility

Species trait values at DRUM: http://hdl.handle.net/1903/21501.
