# Supplementary material for: Recurrent evolution of extreme longevity in bats
Source: Biol Lett. 2019 Apr 10;15(4):20180860. doi: 10.1098/rsbl.2018.0860 (PMC6501359; doi:10.1098/rsbl.2018.0860)
Supplement: Supplemental Methods [file rsbl20180860supp1.docx]

**Expanded Supplemental Methods**

**for "Recurrent Evolution of Extreme Longevity in Bats" by**

**Gerald S. Wilkinson and Danielle M. Adams**

We downloaded data on body mass and longevity for 92 bat species and 804 nonflying placental mammals from AnAge, build 14. To minimize the chance that our analysis might be influenced by underestimates of longevity derived from small sample sizes, we excluded species for which sample sizes were scored below "medium" and data quality was scored as "low" or "questionable." We also excluded one species that was missing from the phylogeny, and revised longevity estimates for four species (*Phyllostomus discolor*, *Desmodus rotundus*, *Pteropus hypomelanus*, and *P. vampyrus*) using new captive records and one species, *Phyllostomus hastatus,* from recapture data [[1](#_ENREF_1)]. We estimated predicted longevity for each bat species from a least squares regression, i.e. log_10_(longevity) = 0.5609 + log_10_(body mass) * 0.1868, using only nonbat placental mammals. We then used a phylogeny [[2](#_ENREF_2)] based on likelihood analysis of 11,465 bp of DNA sequence to reconstruct the longevity quotient (LQ), the ratio of observed to predicted longevity for a nonbat placental mammal of the same body mass, with squared-change parsimony using Mesquite v. 3.6 [[3](#_ENREF_3)]. This method of ancestral state reconstruction for a continous character minimizes the sum of squared-changes along the branches of a phylogenetic tree, as expected under a Brownian motion model of evolution.

To determine which variables explain variation in bat longevity we evaluated linear models using an information-theoretic approach [[4](#_ENREF_4)]. We corrected for common ancestry using phylogenetic generalized least squares (PGLS), as implemented in the R package CAPER [[5](#_ENREF_5)]. We conducted the PGLS analyses on log_10_(longevity), not LQ, using log_10_(body mass) as one of several predictor variables. We did this because LQ is a ratio and, therefore, is not independent of either longevity or body mass. In addition, it is not normally distributed. Using it as the response variable in a linear model is, therefore, undesirable. We predicted that longevity should be influenced by hibernation duration and used the absolute value of the median latitude of the species' range as a proxy for annual temperature and hibernation duration because in rodents hibernation duration increases linearly with mean annual temperature [[6](#_ENREF_6)]. We included an interaction between hibernation and latitude to allow for the possibility that latitude may not affect longevity in species that do not hibernate. We also included a binary variable to indicate whether individuals ever use a cave or cave-like structure and log_10_ (breeding aggregation size) to assess if either of these variables influence mortality risk. We included the primary food source (i.e. animal or plant material) in the diet and the average number of offspring produced per year. Finally, to determine if sexual selection on body size contributes to variation in longevity we used sexual dimorphism in total body length (TL), as measured by log_2_ (male-TL/female-TL), under the assumption that male-biased sexual dimorphism is the result of sexual selection for male competitive ability. Trait values were obtained from the literature, museum collections, personal observation or personal communication. Museum specimen measurements were extracted from online records accessed via VertNet (vertnet.org). Trait values used in this study are available online at DRUM: <http://hdl.handle.net/1903/21501>.

We measured the relative importance of the phylogeny in predicting each trait by calculating Pagel's λ for all continuous variables and D for all binary variables. Pagel's λ measures the degree to which a continuous variable changes in accordance with phylogenetic relationships with λ =1 indicating variation in the trait is consistent with Brownian evolution, and λ = 0 indicating random variation independent of the phylogeny. D measures the degree to which a binary trait changes along the edges of the phylogeny in comparison to random expectations [[7](#_ENREF_7)] with D = -1 indicating all change is predicted by the phylogeny.

We used ten predictors (nine variables plus one two-way interaction) and fit all possible models using phylogenetic generalized least squares [[8](#_ENREF_8)]. We rank-ordered the models by the corrected Akaike information criterion (AICc) and calculated Akaike weights to determine the relative strength of each model. We selected all models within four AICc of the best-fitting model to use for model averaging and estimated weighted coefficients, confidence intervals, and relative importance for each variable [[4](#_ENREF_4), [9](#_ENREF_9)]. Because the interaction between hibernation and latitude had significant influence in all models, to interpret the effects of the remaining variables we split the data according to whether species hibernate or not, and then repeated the analyses described above.

**References**

[1] Wilkinson, G.S., Carter, G.G., Bohn, K.M. & Adams, D.M. 2016 Non-kin cooperation in bats. *Philos. Trans. R. Soc. Lond. B* **371**, 20150095. (doi:10.1098/rstb.2015.0095).

[2] Amador, L.I., Arevalo, R.L.M., Almeida, F.C., Catalano, S.A. & Giannini, N.P. 2018 Bat systematics in the light of unconstrained analyses of a comprehensive molecular supermatrix. *J. Mamm. Evol.* **25**, 37-70. (doi:10.1007/s10914-016-9363-8).

[3] Maddison, W.P. & Maddison, D.R. 2018 Mesquite: a modular system for evolutionary analysis. (3.6 ed.

[4] Burnham, K.P. & Anderson, D.R. 2003 *Model Selection and Multimodel Inference: A Practical Information-theoretic Approach*. Second ed. New York, Springer Science & Business Media; 488 p.

[5] Orme, D. 2013 The CAPER package: comparative analysis of phylogenetics and evolution in R. (R package version 5.

[6] Turbill, C. & Prior, S. 2016 Thermal climate‐linked variation in annual survival rate of hibernating rodents: shorter winter dormancy and lower survival in warmer climates. . *Funct. Ecol.* **30**, 1366-1372. (doi:10.1111/1365-2435.12620).

[7] Fritz, S.A. & Purvis, A. 2010 Selectivity in mammalian extinction risk and threat types: a new measure of phylogenetic signal strength in binary traits. *Conserv. Biol.* **24**, 1042-1051. (doi:10.1111/j.1523-1739.2010.01455.x).

[8] Freckleton, R.P., Harvey, P.H. & Pagel, M. 2002 Phylogenetic analysis and comparative data: a test and review of evidence. *Am. Nat.* **160**, 712-726. (doi:10.1086/343873).

[9] Symonds, M.R. & Moussalli, A. 2011 A brief guide to model selection, multimodel inference and model averaging in behavioural ecology using Akaike’s information criterion. *Behav. Ecol. Sociobiol.* **65**, 13-21.
