# Supplementary material for: Recurrent evolution of extreme longevity in bats
Source: Biol Lett. 2019 Apr 10;15(4):20180860. doi: 10.1098/rsbl.2018.0860 (PMC6501359; doi:10.1098/rsbl.2018.0860)
Supplement: Supplemental Table 1 [file rsbl20180860supp2.docx]

**Supplemental Table 1**

**for "Recurrent Evolution of Extreme Longevity in Bats" by**

**Gerald S. Wilkinson and Danielle M. Adams**

Phylogenetic signal (λ or D) in each variable. Estimates that differ from 0 are indicated in bold.

| Variable | Estimate | CI(λ) or P(D)* |
| --- | --- | --- |
| **Log (longevity)** | **λ = 0.716** | **0.186-0.916** |
| **Log (mass)** | **λ = 0.881** | **0.729-0.959** |
| **\|Median latitude\|** | **λ = 0.774** | **0.506-0.939** |
| Progeny per year | λ = 0.348 | 0.000-0.596 |
| **Sexual dimorphism** | **λ = 0.317** | **0.026-0.784** |
| Log (aggregation size) | λ = 0.165 | 0.000-0.681 |
| **Hibernation** | **D = -0.640** | **0, 0.948** |
| **Cave use** | **D = -0.160** | **0, 0.663** |
| **Diet** | **D = -1.040** | **0, 0.984** |

*****probability of D resulting from random, or Brownian evolution
