# Supplementary material for: Recurrent evolution of extreme longevity in bats
Source: Biol Lett. 2019 Apr 10;15(4):20180860. doi: 10.1098/rsbl.2018.0860 (PMC6501359; doi:10.1098/rsbl.2018.0860)
Supplement: Supplemental Table 2 [file rsbl20180860supp3.docx]

**Supplemental Table 2**

**for "Recurrent Evolution of Extreme Longevity in Bats" by**

**Gerald S. Wilkinson and Danielle M. Adams**

Rank-ordered PGLS models within 4 AICc of the best model for predicting log longevity of bats. Model abbreviations are M = log(mass), H = hibernator (yes, no), L = absolute value of median latitude of species range, C = cave roosting (yes, no), D = sexual dimorphism in total length (log_2_(M-TL/F-TL)), A = log(aggregation size), P = progeny per year, F = diet (plant, animal), S = data source (captive, wild).

| Model | AICc | ΔAICc | Weight | R^2^ |
| --- | --- | --- | --- | --- |
| M+H+L+C+H*L | -84.95 | 0 | 0.39 | 0.69 |
| M+H+L+C+S+H*L | -82.67 | 2.28 | 0.12 | 0.69 |
| M+H+L+C+D+H*L | -82.38 | 2.57 | 0.11 | 0.67 |
| M+H+L+C+F+H*L | -82.38 | 2.57 | 0.11 | 0.67 |
| M+H+L+C+A+H*L | -82.33 | 2.62 | 0.10 | 0.67 |
| M+H+L+C+P+H*L | -82.19 | 2.76 | 0.10 | 0.67 |
| M+H+L+H*L | -81.53 | 3.42 | 0.07 | 0.63 |
