# Supplementary material for: Recurrent evolution of extreme longevity in bats
Source: Biol Lett. 2019 Apr 10;15(4):20180860. doi: 10.1098/rsbl.2018.0860 (PMC6501359; doi:10.1098/rsbl.2018.0860)
Supplement: Supplemental Table 3 [file rsbl20180860supp4.docx]

**Supplemental Table 3**

**for "Recurrent Evolution of Extreme Longevity in Bats" by**

**Gerald S. Wilkinson and Danielle M. Adams**

Model-averaged conditional coefficients using the PGLS models in Supplemental Table 2 for predicting log longevity of bats. Coefficient estimates that differ from 0 are indicated in bold.

| Variable | Estimate (SE) | Importance |
| --- | --- | --- |
| **Intercept** | **0.781 ± 0.076** |  |
| **Log Mass** | **0.269 ± 0.037** | **1.00** |
| \|Median Latitude\| | -0.003 ± 0.002 | 1.00 |
| Hibernation | -0.123 ± 0.126 | 1.00 |
| **Hibernation*Latitude** | **0.013 ± 0.003** | **1.00** |
| Dimorphism | -0.118 ± 0.280 | 0.11 |
| **Cave use** | **-0.113 ± 0.042** | **0.93** |
| Progeny/yr | 0.0003 ± 0.039 | 0.10 |
| Food | -0.022 ± 0.052 | 0.11 |
| Aggregation size | -0.007 ± 0.018 | 0.10 |
| Data source | -0.026 ± 0.052 | 0.12 |
